# Whipple's (Pancreaticoduodenectomy) Surgery: A Five-Year Clinical Experience in Gastrointestinal Surgery

**DOI:** 10.7759/cureus.117562

**Published:** 2026-10-06

**Authors:** Sunil Dhakal, Nimesh Bista, Bibek Neupane, Saurav Karki, Deepak Sharma, Ijen Prajapati, Ganga Sapkota

**Affiliations:** 1 Gastrointestinal Surgery, B.P. Koirala Memorial Cancer Hospital, Bharatpur, NPL; 2 Surgical Gastroenterology, Maharajgunj Medical Campus, Institute of Medicine, Tribhuvan University Teaching Hospital, Kathmandu, NPL; 3 Gastrointestinal Oncosurgery, B.P. Koirala Memorial Cancer Hospital, Bharatpur, NPL; 4 Surgical Gastroenterology, Nepalese Army Institute of Health Sciences, Shree Birendra Hospital, Kathmandu, NPL; 5 Surgery, Patan Academy of Health Sciences, Kathmandu, NPL

**Keywords:** dge, outcomes, pancreaticoduodenectomy, popf, pph

## Abstract

Background: Pancreaticoduodenectomy (PD) is a complex surgical procedure commonly performed for periampullary and pancreatic malignancies. Although perioperative morbidity and mortality have improved over time, outcomes continue to vary across institutions. This study aimed to address the lack of regional data by evaluating the perioperative factors associated with early postoperative outcomes following PD over a five-year period at our institution.

Method: This retrospective study included 83 patients who underwent PD between September 2018 and September 2023 at the Department of Gastrointestinal Surgery at B.P. Koirala Memorial Cancer Hospital, Bharatpur, Nepal. Preoperative, intraoperative, and postoperative variables were collected and analyzed. Postoperative complications were assessed according to standard definitions, and overall postoperative morbidity was quantified using the Comprehensive Complication Index (CCI). Patients were categorized into minor (CCI ≤23) and major (CCI >23) complication groups. Perioperative factors associated with major complications were evaluated using univariate and multivariable logistic regression analyses.

Results: The mean age of the patients was 53.1±11.5 years, and 44 (53%) patients were male. Comorbidities were present in 53 (63.9%) patients. The mean BMI was 23.2±2.67 kg/m², while the median (IQR) preoperative bilirubin level was 6.32 (1.42-11.8) mg/dL, and the mean serum albumin level was 3.47±0.66 g/dL. Ampullary carcinoma was the most common indication for surgery. Stage III disease was the most frequent pathological stage, occurring in 28 (33.7%) patients, followed by stage II disease in 27 (32.5%) patients. The mean operative time was 313±46.5 minutes, and the median (interquartile range (IQR)) intraoperative blood loss was 400 (300-585) mL. Major postoperative complications, defined as a CCI >23, occurred in 24 (28.9%) patients. Postoperative pancreatic fistula (POPF) developed in 26 (31.3%) patients, delayed gastric emptying (DGE) in 31 (37.3%), bile leak in 11 (13.3%), and postpancreatectomy hemorrhage (PPH) in two (2.4%). Overall postoperative mortality was 2.4% (two patients). On univariate analysis, age, preoperative bilirubin, serum albumin, intraoperative blood loss, soft pancreatic texture, POPF, DGE, and bile leak were significantly associated with major postoperative complications. On multivariable analysis, POPF (OR: 4.27; 95% CI: 1.38-13.20; p=0.012), bile leak (OR: 13.08; 95% CI: 2.38-71.80; p=0.003), and soft pancreas (OR: 3.03; 95% CI: 1.02-8.98; p=0.04) were independently associated with major postoperative complications. Age (OR: 1.05; 95% CI: 0.99-1.10; p=0.05) and preoperative bilirubin (OR: 1.54; 95% CI: 0.98-2.41; p=0.05) demonstrated borderline associations.

Conclusion: Our study suggests that soft pancreatic texture showed a strong association with major complications, while older age and higher preoperative bilirubin demonstrated borderline associations. Among the postoperative factors, the occurrence of POPF and bile leak was strongly associated with major postoperative complications. Early identification of these morbidity factors and appropriate optimization and mitigation strategies may help reduce postoperative complications. Prospective studies evaluating morbidity factor modification and optimization, with larger study populations and long-term follow-up, are warranted to validate these findings and improve outcomes after PD.

## Introduction

Pancreaticoduodenectomy (PD), also known as the Whipple procedure, is a complex abdominal operation performed for both malignant and selected benign lesions of the pancreatic head, distal bile duct, duodenum, and periampullary region. It is technically demanding, involving en bloc resection of the pancreatic head, duodenum, distal bile duct, and gallbladder, followed by complex reconstruction with multiple anastomoses [1,2]. Despite a significant reduction in operative mortality from 25-30% in the early era to approximately 2-4% in the modern era, PD continues to be associated with substantial postoperative morbidity, reported in 30-40% of cases [3,4]. The most common complications include postoperative pancreatic fistula (POPF), delayed gastric emptying (DGE), postpancreatectomy hemorrhage (PPH), and intra-abdominal collections [3,4].

PD is commonly performed in our region; however, reported morbidity remains high, ranging from 50% to 60%, while mortality varies between 0% and 11% across published series [1,5,6]. Early postoperative complications are major contributors to postoperative morbidity and mortality and may substantially prolong hospital stay, increase healthcare utilization, and adversely affect subsequent treatment and long-term outcomes [7]. Therefore, identification of perioperative factors associated with postoperative complications is important for early recognition, prevention, and timely management of patients at increased risk. Early postoperative complications are generally defined as deviations from the normal postoperative course occurring within 30 days after surgery [8]. Despite PD being performed in our region, the regional data regarding perioperative factors associated with early postoperative outcomes remain limited. Our institution is one of the major oncology centers in Nepal and manages a substantial volume of patients with pancreatic and periampullary malignancies.

Therefore, this study was undertaken to evaluate the perioperative factors associated with early postoperative outcomes following PD over a five-year period at our institution. The findings are expected to contribute regional outcome data, facilitate comparison with international standards, and identify potentially modifiable factors for quality improvement and the optimization of perioperative care.

## Materials and methods

This retrospective study analyzed data from all patients who underwent PD between September 2018 and September 2023. A total of 83 patients were included. The study was conducted in the Department of Gastrointestinal Surgery at B.P. Koirala Memorial Cancer Hospital in Bharatpur, Chitwan, Nepal. The study protocol was approved by the B.P. Koirala Memorial Cancer Hospital Institutional Review Committee (approval number: 109/2022/2023). All patients underwent preoperative contrast-enhanced computed tomography (CECT) of the abdomen and pelvis with a pancreatic protocol. Preoperative biliary drainage was performed in patients with cholangitis or in those with anticipated delay to surgery specifically for those requiring neoadjuvant therapy. Neoadjuvant therapy was administered in cases of borderline resectable pancreatic cancer.

Surgical aspect

All procedures were performed using an open surgical approach by a single surgeon. Pancreatic texture was assessed intraoperatively by the operating surgeon based on manual palpation and categorized as soft or firm according to the surgeon's subjective assessment. All pancreatic resections were performed using monopolar diathermy. Pancreatojejunostomy (PJ) was constructed using the modified Blumgart technique, with 5-0 polydioxanone sutures for duct-to-mucosa anastomosis. Hepaticojejunostomy (HJ) was performed using an interrupted technique with 4-0 polydioxanone sutures. Gastrojejunostomy (GJ) was created using a linear stapler and 4-0 polydioxanone suture. Intraoperative inotropic support was initiated when the mean arterial pressure (MAP) remained below 65 mmHg despite adequate fluid resuscitation. This decision was based primarily on the hemodynamic response rather than a predefined volume of blood loss and was made according to institutional practice.

Outcome analysis

PD-specific postoperative complications were defined according to the International Study Group for Pancreatic Surgery (ISGPS). POPF was defined and graded according to the ISGPS 2016 criteria as a drain fluid amylase level >3 times the upper limit of the institutional normal serum amylase value, measured on or after postoperative day (POD) 3. Grade B and grade C were considered clinically relevant pancreatic fistulas, while grade A was reclassified as a biochemical leak [9]. PPH was defined based on timing (early or late), location (intraluminal or extraluminal), and severity (grades A-C) [10]. DGE was defined as the need for nasogastric decompression after POD 3 or intolerance to oral intake in the absence of mechanical obstruction. It was classified into grades A, B, and C based on clinical severity and duration [11]. Bile leak was defined as drain bilirubin >3 times the serum bilirubin on or after POD 3 [12]. Chyle leak was defined as milky-colored fluid output from the drain or wound after POD 3 with triglyceride content ≥110 mg/dL [13]. The Comprehensive Complication Index (CCI) was used to evaluate the postoperative complications; a score >23 was regarded as major complications and ≤23 as minor complications [14]. Demographic characteristics, intraoperative variables, postoperative outcomes, complications, and histopathological findings were recorded and analyzed.

Data analysis

Categorical variables were expressed as frequencies and percentages and compared using the chi-squared test or Fisher's exact test, as appropriate. Continuous variables were assessed for normality and summarized as mean±standard deviation (SD) for normally distributed data and median with interquartile range (IQR) for non-normally distributed data. Normally distributed continuous variables were compared using Student’s t-test, whereas non-normally distributed variables were compared using the Mann-Whitney U test. Where appropriate, the magnitude of between-group differences was also reported as mean difference for normally distributed variables or median difference for non-normally distributed variables, together with the corresponding 95% confidence interval (CI).

Univariable and multivariable binary logistic regression analyses were performed to identify factors independently associated with major postoperative complications, defined as a CCI >23. Prior to regression analysis, correlations between potential predictor variables were assessed using Pearson's correlation coefficient for normally distributed continuous variables and Spearman's rank correlation coefficient for non-normally distributed variables. Highly correlated variables were not simultaneously included in the multivariable model to minimize multicollinearity. Multicollinearity was further assessed using the variance inflation factor (VIF), with a VIF <3 considered acceptable.

Variables showing a statistically significant association with major postoperative complications in the univariable analysis (p<0.05) were considered for inclusion in the multivariable logistic regression model, along with clinically relevant variables where appropriate. Before analysis of the final model, foundational assumptions of binomial logistic regression will be checked. Variables that do not follow a normal distribution will undergo logarithmic transformation, where appropriate, to improve the approximation of normality. The transformed values will be used in subsequent univariable and multivariable analyses. A binary logistic regression model was constructed because the dependent variable, CCI >23, was dichotomous. The final model was limited to a maximum of three predictor variables to maintain model stability given the sample size and number of outcome events.

Standardized regression estimates (β) were also reported where applicable. A standardized estimate represents the change in the dependent variable associated with a one-standard-deviation change in a continuous predictor and allows the relative contribution of predictors measured on different scales to be compared. For categorical predictors, effect estimates were interpreted using the corresponding odds ratios (OR) relative to the reference category. OR, 95% CI, and p-values were reported.

Model assumptions, including the absence of problematic multicollinearity and the suitability of the predictor variables for binary logistic regression, were assessed before the interpretation of the final model. Model fit was also evaluated using appropriate goodness-of-fit measures and reported with Cox and Snell's pseudo R-square (R2CS).

Statistical analyses were performed using IBM SPSS Statistics for Windows, V. 30.0 (IBM Corp., Armonk, NY, USA). All tests were two-sided, and a p-value of <0.05 was considered statistically significant.

## Results

A total of 83 patients underwent PD over the five-year study period. The mean age was 53.1±11.5 years, with males comprising 53% and females 47% of the cohort. The mean body mass index (BMI) was 23.2±2.67 kg/m². Comorbidities were present in 63.9% of patients, with diabetes mellitus being the most common (49.3%), followed by hypertension (38.5%). Ampullary carcinoma was the most frequent indication for PD, observed in 38.6% of patients, followed by distal cholangiocarcinoma and pancreatic ductal adenocarcinoma in 21.7% of patients each. Preoperative biliary drainage was performed in 16 patients. Cholangitis was the indication in 13 patients, while in three patients, it was performed because of a delay in definitive surgery. All eight patients who received neoadjuvant therapy underwent preoperative biliary drainage. Neoadjuvant therapy was administered in 12 patients (14.5% of patients), while the remaining patients underwent upfront surgery. The preoperative characteristics are presented in Table 1.

**Table 1:** Preoperative characteristics Values are expressed as mean±SD, median (IQR), and numbers (percentages). ASA: American Society of Anesthesiologists; BMI: body mass index; DM: diabetes mellitus; COPD: chronic obstructive pulmonary disease; CEA: carcinoembryonic antigen; CA19-9: carbohydrate antigen 19-9; IQR: interquartile range

Preoperative variables	Total (N=83)
Age (mean±SD), years	53.1±11.5
Male, n (%)	44 (53%)
Female, n (%)	39 (47%)
ASA grade	
1, n (%)	40 (48.2%)
2, n (%)	33 (39.8%)
3, n (%)	10 (12%)
BMI (mean±SD), kg/m^2^	23.2±2.67
Comorbidity, n (%)	53 (63.9%)
DM, n (%)	41 (49.3%)
Hypertension, n (%)	32 (38.5%)
COPD, n (%)	4 (4.8%)
Hypothyroidism, n (%)	8 (9.6%)
Preoperative bilirubin, median (IQR), mg/dL	6.32 (1.42-11.8)
Preoperative albumin (mean±SD), mg/dL	3.47±0.66
Preoperative biliary drainage, n (%)	16 (19.3%)
Preoperative CEA (median±SD), ng/mL	4.41±20.05
Preoperative CA19-9 (median±SD), U/mL	38.5±337
Indication of surgery	
Ampullary carcinoma, n (%)	32 (38.6%)
Distal cholangiocarcinoma, n (%)	18 (21.7%)
Pancreatic ductal adenocarcinoma, n (%)	18 (21.7%)
Chronic pancreatitis with head mass, n (%)	7 (8.4%)
Duodenal carcinoma, n (%)	4 (4.8%)
Others, n (%)	4 (4.8%)
Neoadjuvant therapy, n (%)	12 (14.5%)

The mean duration of surgery was 313±46.5 minutes, with a median intraoperative blood loss of 400 with an IQR of 300-585 mL. On intraoperative assessment, 54.2% of patients had a soft pancreas, while 45.8% had a firm pancreas. The intraoperative characteristics are presented in Table 2.

**Table 2:** Intraoperative characteristics Values are expressed as mean±SD, median (IQR), and numbers (percentages). IQR: interquartile range

Intraoperative variables	Total (N=83)
Blood loss, median (IQR), mL	400 (300-585)
Duration of surgery (mean±SD), minutes	313±46.5
Inotropes required, n (%)	28 (33.7%)
Texture of pancreas	
Soft, n (%)	45 (54.2%)
Firm, n (%)	38 (45.8%)

Postoperatively, the median length of hospital stay was 13 days with an IQR of 11-17.5 days. The median CCI was 12.2±18.67, and 24 (28.9%) patients developed major complications. POPF occurred in 26 patients (31.3%), with the majority being biochemical leak and grade B present in 11 patients each, while four patients developed grade C POPF, in which three patients developed organ failure and one patient died because of sepsis. DGE was observed in 31 patients (37.3%); the most common grade was grade B present in 15 patients, followed by grade A in 14 patients and grade C in two patients. PPH was observed in two patients, which was both early and extraluminal types. Both patients required re-exploration in which bleeding was from the omental vessels in one patient and in another from the pancreatic stump. Mortality was observed in one patient developing PPH. Bile leak was present in 11 patients, with 10 cases classified as grade A and one case as grade B; no grade C bile leaks were observed. Surgical site infection occurred in 51.8% of patients. The overall mortality rate was 2.4%, which occurred in two patients. On histopathological examination, the majority (33.7%) of patients had stage III disease, followed by stage II in 32.5% of patients and stage I in 20.4% of patients. The postoperative characteristics are presented in Table 3.

**Table 3:** Postoperative characteristics Values are expressed as mean±SD, median (IQR), and numbers (percentages). POPF: postoperative pancreatic fistula; DGE: delayed gastric emptying; PPH: postpancreatectomy hemorrhage; SSI: surgical site infection; CCI: Comprehensive Complication Index; IQR: interquartile range

Postoperative variable	Total (N=83)
POPF, n (%)	26 (31.3%)
Biochemical leak, n (%)	11 (13.2%)
Grade B, n (%)	11 (13.2%)
Grade C, n (%)	4 (4.8%)
DGE, n (%)	31 (37.3%)
Grade A, n (%)	14 (16.9%)
Grade B, n (%)	15 (18.1%)
Grade C, n (%)	2 (2.4%)
PPH, n (%)	2 (2.4%)
Bile leak, n (%)	11 (13.3%)
Grade A, n (%)	10 (12.1%)
Grade B, n (%)	1 (1.2%)
Grade C, n (%)	0
SSI, n (%)	43 (51.8%)
CCI (median±SD)	12.2±18.67
Major complication, n (%)	24 (28.9%)
Length of hospital stay, median (IQR) days	13 (11-17.5)
Mortality, n (%)	2 (2.4%)
Stage, n (%)	72 (86.7%)
I, n (%)	17 (20.4%)
II, n (%)	27 (32.5%)
III, n (%)	28 (33.7%)

Univariate analysis and comparison of patient characteristics and outcomes between CCI ≤23 and CCI >23

The 83 patients were divided into two groups according to the CCI: CCI ≤23 was present in 59 patients, representing the minor complication group, and CCI >23 was present in 24 patients, representing the major complication group. The mean age was significantly higher in the CCI >23 group (p=0.022), with a greater proportion of elderly patients in the major complication group. There was no significant difference between the two groups in terms of sex, BMI, or the presence of comorbidities. The preoperative serum bilirubin level was significantly higher in the CCI >23 group with a median of 9.08 and an IQR of 4.25-15.8 mg/dL. However, there was no significant difference in the proportion of patients undergoing preoperative biliary drainage between the groups. The mean serum albumin was 3.24±0.64 g/dL significantly lower in the CCI >23 group, suggesting a higher prevalence of preoperative malnutrition among patients who developed major complications. There was no significant difference between the groups regarding tumor distribution, disease stage, or American Society of Anesthesiologists (ASA) classification. The univariate analysis comparison is presented in Table 4.

**Table 4:** Univariate analysis of preoperative characteristics Values are expressed as mean±SD, median (IQR), and numbers (percentages). *P-values were calculated using Student's t-test and reported as test statistic (t), degrees of freedom (df), and mean difference (MD) (95% CI). **P-values were calculated using the chi-squared test and reported as test statistic (χ^2^), degrees of freedom (df), and odds ratio (OR) (95% CI). ***P-values were calculated using the Mann-Whitney U test due to non-normal distribution and reported as test statistic (U), Z-score, and mean difference (MD) (95% CI). ****P-values were calculated using Fisher's exact test (when cell count is <5) and reported as test statistic (t), degrees of freedom (df), and odds ratio (OR) (95% CI) wherever applicable. BMI: body mass index; ASA: American Society of Anesthesiologists; IQR: interquartile range

Variables		Total (N=83)	CCI ≤23 (n=59)	CCI >23 (n=24)	P-value	Test statistic	Df/Z-score	MD (95% CI)/OR (95% CI)
Age (mean±SD), years		53.1±11.5	51.3±10.7	57.6±12.4	0.022*	t=-2.33	81	-6.33 (-11.7, 0.92)
Sex	Male, n (%)	44 (53%)	30 (50.8%)	14 (58.3%)	0.53**	χ^2^=0.38	1	0.73 (0.28, 1.93)
	Female, n (%)	39 (47%)	29 (49.2%)	10 (41.7%)				
BMI (mean±SD), kg/m^2^		23.2±2.67	23.4±2.55	22.5±2.90	0.17*	t=1.38	81	0.88 (-0.39, 2.17)
Comorbidities	Yes, n (%)	53 (63.9%)	35 (59.3%)	18 (75%)	0.14**	χ^2^=1.82	1	0.48 (0.16- 1.40)
	No, n (%)	30 (36.1%)	24 (40.6%)	6 (25%)				
Preoperative bilirubin, median (IQR), mg/dL		6.32 (1.42-11.8)	4.77 (0.89-11.3)	9.08 (4.25-15.8)	0.04***	U=506	-2.03	-3.26 (-7.03, -0.08)
Preoperative albumin (mean±SD), mg/dL		3.47±0.66	3.57±0.65	3.24±0.64	0.03*	t=2.10	81	0.33 (0.01, 0.64)
Use of neoadjuvant	Yes, n (%)	12 (14.5%)	10 (16.9%)	2 (8.3%)	0.49**	χ^2^=1.02	1	2.24 (0.45- 11.1)
	No, n (%)	71 (85.5%)	49 (83.1%)	22 (91.7%)				
Preoperative drainage	Yes, n (%)	16 (19.3%)	12 (20.3%)	4 (16.6%)	1.00****	-	-	1.28 (0.36, 4.44)
	No, n (%)	67 (80.7%)	47 (79.7%)	20 (83.4%)				
Site of tumor	Ampulla	32 (38.6%)	22 (37.3%)	10 (41.6%)	0.13****	-	-	-
	Chronic pancreatitis	7 (8.4%)	7 (11.9%)	0				
	Distal cholangiocarcinoma	18 (21.7%)	9 (15.2%)	9 (37.5%)				
	Duodenum	4 (4.8%)	3 (5.1%)	1 (4.2%)				
	Head of pancreas	18 (21.7%)	15 (25.4%)	3 (12.5%)				
	Others	4 (4.8%)	3 (5.1%)	1 (4.2%)				
ASA grading	ASA I, n (%)	40 (48.2%)	30 (50.8%)	10 (41.7%)	0.1****	-	-	-
	ASA II, n (%)	33 (39.8%)	25 (42.4%)	8 (33.3%)				
	ASA III, n (%)	10 (12%)	4 (6.8%)	6 (25%)				
Stage of tumor	Stage I	17 (20.4%)	15 (25.4%)	2 (8.3%)	0.09****	-	-	-
	Stage II	27 (32.5%)	15 (25.4%)	12 (50%)				
	Stage III	28 (33.7%)	18 (30.5%)	10 (41.7%)				

The mean duration of surgery in the major complication group was 321±51.6 minutes, which was longer than that in the CCI ≤23 group (310±44.4 minutes); however, this difference was not statistically significant. The intraoperative blood loss was higher in the CCI >23 group with a median of 571 and an IQR of 350-725 mL compared with the CCI ≤23 group which had a median of 400 and an IQR of 250-500 mL. Similarly, the use of inotropes and the presence of a soft pancreatic texture were significantly higher in the major complication group compared with the CCI ≤23 group. The univariate analysis of intraoperative characteristics is presented in Table 5.

**Table 5:** Univariate analysis of intraoperative characteristics Values are expressed as mean±SD, median (IQR), and numbers (percentages). *P-values were calculated using Student's t-test and reported as test statistic (t), degrees of freedom (df), and mean difference (MD) (95% CI). **P-values were calculated using the chi-squared test and reported as test statistic (χ^2^), degrees of freedom (df), and odds ratio (OR) (95% CI). ***P-values were calculated using the Mann-Whitney U test due to non-normal distribution and reported as test statistic (U), Z-score, and mean difference (MD) (95% CI). IQR: interquartile range

Variables		Total	CCI ≤23	CCI >23	P-value	Test statistic	Df/Z-score	MD (95% CI)/OR (95% CI)
Duration of surgery		313±46.5	310±44.4	321±51.6	0.34*	t=-0.95	81	-10.8 (-33.2, 11.6)
Blood loss		400 (300-585)	400 (250-500)	571 (350-725)	0.01***	U=470	Z-score=-2.39	-150 (-250, 0)
Inotropes used	Yes	28 (33.7%)	16 (27.1%)	12 (50%)	0.04**	χ^2^=4	1	2.69 (1.00, 7.19)
	No	55 (66.3%)	43 (72.9%)	12 (50%)				
Texture of pancreas	Hard	38 (45.8%)	32 (54.2%)	6 (25%)	0.01**	χ^2^=5.88	1	0.28 (0.09, 0.80)
	Soft	45 (54.2%)	27 (45.8%)	18 (75%)				

The postoperative hospital stay was significantly longer in the major complication group, with a median of 17.5 and an IQR of 12.8-20.8 days, compared with a median of 12 and an IQR of 11-15 days in the CCI ≤23 group. Similarly, the incidence of PPH, POPF, and DGE was significantly higher in the major complication group. The univariate analysis of postoperative characteristics is presented in Table 6.

**Table 6:** Univariate analysis of postoperative characteristics Values are expressed as mean±SD, median (IQR), and numbers (percentages). *P-values were calculated using the Mann-Whitney U test due to non-normal distribution and reported as test statistic (U), Z-score, and mean difference (MD) (95% CI). **P-values were calculated using the chi-squared test and reported as test statistic (χ^2^), degrees of freedom (df), and odds ratio (OR) (95% CI). ***P-values were calculated using Fisher's exact test (when cell count is <5) and reported as test statistic (t), degrees of freedom (df), and odds ratio (OR) (95% CI) (wherever applicable). POPF: postoperative pancreatic fistula; DGE: delayed gastric emptying; IQR: interquartile range

Variables		Total	CCI ≤23	CCI >23	P-value	Test statistics	Df/Z-score	MD (95% CI)/OR (95% CI)
Postoperative hospital stay		13 (11-17.5)	12 (11-15)	17.5 (12.8-20.8)	<0.001*	U=368	-3.42	-4 (-7.00, -2.00)
POPF	Yes	26 (31.3%)	12 (20.3%)	14 (58.3%)	<0.001**	χ^2^=11.4	1	5.48 (1.96, 15.4)
	No	57 (68.7%)	47 (79.7%)	10 (41.7%)				
Bile leak	Yes	11 (13.3%)	2 (3.4%)	9 (37.5%)	<0.001***	-	-	17.1 (3.34, 87.7)
	No	72 (86.7%)	57 (96.6%)	15 (62.5%)				
DGE	Yes	31 (37.3%)	15 (25.4%)	16 (66.7%)	<0.001**	χ^2^=12.4	1	5.86 (2.09, 16.45)
	No	52 (62.7%)	44 (74.6%)	8 (33.3%)				

In multivariable logistic regression analysis, soft pancreatic texture, POPF, and bile leak were independently associated with major postoperative complications. Soft pancreatic texture was associated with approximately threefold higher odds of major complications (OR: 3.03; 95% CI: 1.02-8.98; p=0.04), while POPF was associated with more than fourfold higher odds (OR: 4.27; 95% CI: 1.38-13.20; p=0.012). Bile leak demonstrated the strongest association, with approximately 13-fold higher odds of major complications (OR: 13.08; 95% CI: 2.38-71.80; p=0.003). Age and preoperative bilirubin showed borderline associations, whereas preoperative albumin and inotrope use were not significantly associated with major complications. The multivariable logistic regression is presented in Tables 7-9.

**Table 7:** Multivariate logistic regression analysis of preoperative variables In the univariate analysis, age (p=0.022), preoperative bilirubin (p=0.04), and preoperative albumin (p=0.03) showed statistical significance. Thus, these variables were then inserted into the correlation matrix by calculating the correlation coefficient using Pearson's correlation for normally distributed data and Spearman's correlation for skewed data. Correlation analysis demonstrated no significant correlation between these variables. Thus, this allows us to include these variables eligible for multivariate analysis. To evaluate the preoperative factors influencing the complications, a binomial logistic regression model was constructed. All predictors were entered into the model simultaneously (the "Enter" method) to assess their combined and individual effects on the outcome. Before analysis of the final model, foundational assumptions of binomial logistic regression were verified. The model met the assumption of absence of repeated measures and paired data; no multicollinearity was seen with variance inflation factor (VIF) <3, and the sample size was adequate for three predictor variables to be used. In this model, the assumption of normality was not met for preoperative bilirubin levels. Therefore, preoperative bilirubin was logarithmically transformed, and the log-transformed values were used in the analysis. The correlation coefficient was calculated using Pearson's correlation for normally distributed data and Spearman's correlation for skewed data. The binomial logistic regression model was tested using a chi-squared test which showed χ^2^= 12.6 with 3 degrees of freedom (df), a p-value of 0.006, and Cox and Snell pseudo R2=0.141. Preoperative total bilirubin was logarithmic-transformed prior to entry to satisfy the linear assumptions of log-odds. Multicollinearity among predictors was ruled out (VIF <3.0; tolerance >0.20).

Variable	Standardized estimate	OR (95% CI)	P-value
Age	0.02	1.05 (0.99-1.10)	0.05
Preoperative bilirubin	0.22	1.54 (0.98-2.41)	0.05
Preoperative albumin	-0.42	0.55 (0.2-1.27)	0.16

**Table 8:** Multivariate logistic regression analysis of intraoperative variables In the univariate analysis, blood loss (p=0.01), inotropes used (p=0.04), and texture of pancreas (p=0.01) showed statistical significance. Thus, these variables were then inserted into a correlation matrix by calculating the correlation coefficient using Pearson's correlation for normally distributed data and Spearman's correlation for skewed data. But the correlation coefficient between inotropes used and blood loss was found to be 0.74 which showed that these variables are correlated with each other. Thus, we will use only one variable that is inotropes used for the multivariate model as using both variables will destabilize the model. To evaluate the preoperative factors influencing the complications, a binomial logistic regression model was constructed. All predictors were entered into the model simultaneously (the "Enter" method) to assess their combined and individual effects on the outcome. The model met the assumption of absence of repeated measures and paired data; no multicollinearity was seen with variance inflation factor (VIF) <3, and the sample size was adequate for three predictor variables to be used. In this model, the assumption of normality was not met for blood loss. Therefore, blood loss was logarithmically transformed, and the log-transformed values were used in the analysis. As the correlation coefficient between inotropes used and blood loss was found to be 0.74 which showed that these variables are correlated with each other, we used the broader variable, use of inotropes, for our model. The binomial logistic regression model was tested using a chi-squared test which showed χ^2^=8.20 with 2 degrees of freedom (df), a p-value of 0.017, and Cox and Snell pseudo R2=0.094. Intraoperative blood loss was excluded from multivariable modeling due to high collinearity with inotrope requirement (r=0.74; p<0.001), retaining inotrope use as the primary systemic indicator. Multicollinearity among predictors was ruled out (VIF <3.0; tolerance >0.20).

Variable		Standardized estimate	OR (95% CI)	P-value
Use of inotropes	Not used	1	-	-
	Used	0.52	2.13 (0.76-5.96)	0.14
Texture of pancreas	Hard	1	-	-
	Soft	0.55	3.03 (1.02-8.98)	0.04

**Table 9:** Multivariate logistic regression analysis of postoperative variables In the univariate analysis, amylase at day 3 (p<0.001) and day 5 (p=0.016), POPF and grade (p=<0.001), and bile leak (p<0.001) showed statistical significance. Thus, these variables were then inserted into a correlation matrix by calculating the correlation coefficient using Pearson's correlation for normally distributed data and Spearman's correlation for skewed data. This showed that there was a correlation between drain amylase on the third and fifth days. Thus, drain amylase at day 3 was taken as it would be the earliest predictor. Correlation analysis demonstrated no significant correlation between other variables. Thus, this allows us to include these variables eligible for multivariate analysis. To evaluate the postoperative factors influencing the complications, a binomial logistic regression model was constructed. All predictors were entered into the model simultaneously (the "Enter" method) to assess their combined and individual effects on the outcome. Before analysis of the final model, foundational assumptions of binomial logistic regression were verified. The use of both POPF grade and drain amylase at day 3 destabilizes the model. As POPF grade is a direct clinical indicator derived from drain amylase (thus adding both of these in the multivariate model will increase multicollinearity), we discarded the drain amylase at day 3 from the multivariate model. Thus, we kept POPF and grade and bile leak grade in the multivariate model. The model met the assumption of absence of repeated measures and paired data; no multicollinearity was seen with variance inflation factor (VIF) <3, and the sample size was adequate for three predictor variables to be used. The binomial logistic regression model was tested using a chi-squared test which showed χ^2^=22.1 with 2 degrees of freedom (df), a p-value of <0.001, and Cox and Snell pseudo R2=0.234. Multicollinearity among predictors was ruled out (VIF <3.0; tolerance >0.20).

Variable		Standardized estimate	OR (95% CI)	P-value
POPF and grade	No	1	-	-
	Yes	0.57	4.27 (1.38-13.20)	0.012
Bile leak grade	No	1	-	-
	Yes	0.86	13.08 (2.38-71.80)	0.003

## Discussion

PD is performed for both malignant and benign lesions involving the pancreatic head, distal biliary tract, ampulla, and duodenum. The majority of indications are malignant. Pancreatic ductal adenocarcinoma remains the most common indication for PD, accounting for approximately 56-66% of cases in major series [15]. Historically, ampullary carcinoma was considered a relatively uncommon indication; however, more recent studies have reported ampullary carcinoma in approximately 15-25% of PD, as nearly 80% of ampullary carcinomas are resectable at presentation [15]. In our study, ampullary carcinoma was the most common indication for PD, observed in 38.6% of patients. A similar higher incidence, reaching up to 53%, has been reported by Pandey et al. from the same geographical region [1]. With the increasing use of multimodality treatment strategies, 12 patients who presented with locally advanced disease underwent preoperative neoadjuvant therapy according to guideline recommendations [16]. Most patients in our study underwent upfront surgery, likely because ampullary carcinoma constituted a significant proportion of cases, and these tumors are frequently resectable at presentation.

Globally, there is a growing trend toward minimally invasive approaches for PD, aimed at reducing surgical trauma. Laparoscopic PD has been associated with reduced intraoperative blood loss; however, no significant difference in overall morbidity has been demonstrated in comparative studies [17]. A recent meta-analysis reported that the operative duration of laparoscopic PD is significantly longer than that of open surgery by approximately 91 minutes, while mean blood loss is reduced by approximately 97 mL in laparoscopic procedures [18]. In the present study, all procedures were performed using the open technique. The mean operative time was 313±46.5 minutes, and the median blood loss was 400 with an IQR of 300-585 mL. Published benchmark data from high-volume early and mid-era series of open PD report operative times of approximately 300-360 minutes and blood loss of around 500 mL [19]. Our outcomes fall within acceptable benchmark limits. These findings suggest that operative efficiency and intraoperative blood loss in our series are comparable to established standards.

POPF remains a significant cause of postoperative morbidity, frequently leading to complications such as hemorrhage and sepsis. In 2016, ISGPS redefined grade A POPF as a "biochemical leak" because of its lack of clinical significance, while grades B and C were classified as clinically relevant POPF (CRPOPF) based on the requirement for therapeutic interventions, including radiological or endoscopic procedures and re-operation [9]. A meta-analysis by Eshmuminov et al. reported an incidence of CRPOPF after PD of approximately 15% [20]. In our series, CRPOPF was observed in 18% of patients, with one associated mortality, which is comparable to the 17% incidence reported in a study conducted within the same region [21]. CRPOPF has been associated with obesity, main pancreatic duct (MPD) diameter <3 mm, and soft pancreatic texture [22]. In our series, 54.2% of patients had a soft pancreatic texture, among whom nine developed CRPOPF, whereas six patients with a firm pancreas developed CRPOPF. Most patients in our study had a BMI within the normal range, with a mean BMI of 23.2±2.67. DGE contributes to prolonged hospital stay, increased healthcare costs, and reduced quality of life. Its reported incidence ranges from 19% to 57% of patients [23]. The risk factors were preoperative biliary drainage, soft pancreas, and older age [23]. In our study, two of these factors, soft pancreatic texture and older age, were more frequently observed among patients who developed major postoperative complications. In our series, DGE was observed in 37.3% of patients, among whom two patients developed grade III DGE requiring nasogastric tube placement for three weeks.

Bile leak after PD has been reported in up to 8% of patients and is generally associated with lower morbidity and mortality compared with other postoperative complications [24]. However, it can predispose to intra-abdominal collections and abscess formation, thereby prolonging hospital stay. Reported risk factors for biliary fistula include extensive skeletonization of the bile duct, creation of the anastomosis on the common bile duct rather than the common hepatic duct, and small bile duct diameter. Intraoperative placement of surgical drains near the anastomotic site facilitates early detection of bile leakage [24]. Recently, there has been a trend toward selective drain placement and early drain removal [24]. In our practice, a single right subhepatic drain is routinely placed to drain the HJ and PJ sites. The drain is usually removed after POD 5 if the drain amylase level is normal. In our study, bile leak was observed in 13.3% of patients, all diagnosed based on drain bilirubin >3 times the serum bilirubin [12]. Most patients were managed conservatively, while one patient required the escalation of antibiotic therapy. The relatively high bile-leak rate in our study may be partly explained by the high proportion of ampullary carcinomas (38.6%), which often present early with biliary obstruction and undergo surgery before marked ductal dilatation [25,26]. This hypothesis of high bile leak in our study requires the prospective evaluation of bile duct diameter, obstruction duration, biliary drainage, tumor location, and HJ-related technical factors. The relatively high incidence of surgical site infection (51.8%) in our study may be multifactorial. POPF (31.3%), bile leak (13.3%), and soft pancreatic texture (54.2%) were relatively frequent in our study population. Postoperative bile leak and pancreatic fistula may contribute to local contamination and infectious complications [27,28]. The high surgical site infection rate observed in our study may therefore reflect the combined effects of these patients and procedure-related factors.

PPH is not the most common postoperative complication after PD, but it is among the most feared because of its high mortality. The reported incidence ranges from 1% to 8%, while mortality rates may range from 11% to 38% [29]. Early PPH, defined as bleeding occurring within 24 hours after surgery, has a reported incidence of 2-5% and is mainly related to failure of hemostasis [29]. The bleeding site in early PPH is most commonly the mesenteric region, pancreatic surface, or arterial bed, and the bleeding is usually extraluminal. Approximately half of early PPH cases originate from the mesentery and occur more frequently following open surgery [25]. In our study, PPH was reported in two patients, and both cases were early PPH. One patient had bleeding from the mesentery, while the other had bleeding from the pancreatic surface. Both patients required re-exploration, and one patient subsequently died due to postoperative sepsis. The overall mortality in our study was 2.4%, which is comparable to the mortality rate of less than 3% reported in high-volume centers [23].

Our study has several limitations. It is a single-center retrospective study with some missing data, including preoperative clinical characteristics and pancreatic duct diameter. In addition, long-term outcomes were not evaluated, which is another important limitation of the study.

## Conclusions

Our study suggests that major postoperative complications following PD in our region are associated with preoperative, intraoperative, and postoperative factors. Soft pancreatic texture showed a strong association with major complications, while older age and higher preoperative bilirubin demonstrated borderline associations. Among the postoperative factors, the occurrence of POPF and bile leak was strongly associated with major postoperative complications. Early identification of these morbidity factors and appropriate optimization and mitigation strategies may help reduce postoperative complications. Prospective studies evaluating morbidity factor modification and optimization, with larger study populations and long-term follow-up, are warranted to validate these findings and improve outcomes after PD.
